# Accelerating Aerobic Sludge Granulation by Adding Dry Sewage Sludge Micropowder in Sequencing Batch Reactors

**DOI:** 10.3390/ijerph120810056

**Published:** 2015-08-21

**Authors:** Jun Li, Jun Liu, Danjun Wang, Tao Chen, Ting Ma, Zhihong Wang, Weilong Zhuo

**Affiliations:** 1 College of Biological and Environmental Engineering, Zhejiang University of Technology, No.18 Chao Wang Road, Hangzhou 310014, China; E-Mail: liujun521282@sina.com; 2 College of Civil Engineering and Architecture, Zhejiang University of Technology, No.18 Chao Wang Road, Hangzhou 310014, China; E-Mails: wangdanjun0509@163.com (D.W.); suimofengge-martin@163.com (T.C.); ctaoaaa@gmail.com (T.M.); 3 Zhejiang Zone King Engineering Technology Co. Ltd., 14B, Hangzhou 310000, China; E-Mails: wzh@zone-king.com (Z.W.); zwl@zone-king.com (W.Z.)

**Keywords:** aerobic granulation, granule, filamentous bacteria, micropowder, dry sewage sludge

## Abstract

Micropowder (20–250 µm) made from ground dry waste sludge from a municipal sewage treatment plant was added in a sequencing batch reactor (R2), which was fed by synthetic wastewater with acetate as carbon source. Compared with the traditional SBR (R1), aerobic sludge granulation time was shortened 15 days in R2. Furthermore, filamentous bacteria in bulking sludge were controlled to accelerate aerobic granulation and form large granules. Correspondingly, the SVI decreased from 225 mL/g to 37 mL/g. X-ray Fluorescence (XRF) analysis demonstrated that Al and Si from the micropowder were accumulated in granules. A mechanism hypotheses for the acceleration of aerobic granulation by adding dry sludge micropowder is proposed: added micropowder acts as nuclei to induce bacterial attachment; dissolved matters from the micropowder increase abruptly the organic load for starved sludge to control overgrown filamentous bacteria as a framework for aggregation; increased friction from the movement of micropowder forces the filaments which extend outwards to shrink for shaping granules.

## 1. Introduction

Aerobic granular sludge is a promising biotechnology in wastewater treatment. It has some advantages, including high biomass concentration, fast settling velocity and compact structure [1,2,3]. The earlier work demonstrated that shortening settling time, increasing organic loading rate (OLR), extending starvation period and increasing shear force were beneficial to enhance aerobic granulation [4].

Recently, rapid aerobic granulation has received more attention. Some researchers tried to accelerate the aerobic granulation according to mechanism of “nuclei”. The nuclei hypothesis theory was first proposed by Lettinga *et al*. [5]. This theory suggests that the formation of granules is similar to a crystallization process. Coal ash, activated carbon, calcium chloride and coagulants commonly act as nuclei for anaerobic or aerobic granulation. It was reported that divalent metal ions such as Ca^2+^, Mg^2+^ and Fe^2+^, played an important role in aerobic granulation [6,7,8,9,10]. These results showed that the use of inorganic matters as nuclei accelerated microbial aggregation, enhanced granule strength, decreased the time of granulation and improved the stability. In addition, effects of zero-valent iron (ZVI) on anaerobic granular sludge [11] and aerobic granules [12] were observed. Similarly, adding fragments of granules [13,14] and granular activated carbon (GAC) [15] had a positive on accelerating sludge granulation.

However, the purchase of materials such as Ca^2+^, Mg^2+^, Fe^2+^ or GAC increases the cost of running wastewater treatment. A novel method involving adding dry waste sludge micropowder to accelerate aerobic granulation was proposed. The characteristics of micropowder are somehow different from the matters mentioned above due to achieve the goal of recycling and reusing waste sludge, making it an eco-friendly wastewater treatment. The aim of this work is to investigate the effect of acceleration aerobic granulation in the SBRs by adding micron-sized powder made of residual dry sludge from a municipal sewage treatment plant.

## 2. Materials and Methods

### 2.1. Experimental Set up and Operation

Two identical column SBRs (100 cm in height and 9 cm in diameter) with a working volume of 4 L were used for cultivating aerobic granules. The volumetric exchange ratio was 50%. The SBR without micropowder addition was marked R1, another reactor with micropowder addition was marked R2.

Activated sludge with SVI of 167 mL/g from a municipal sewage treatment plant and bulking sludge with SVI of 225 mL/g from a reactor were used as inoculums, respectively. In this study, the volume of inoculum was 500 mL. The reactors were fed with the acetate-containing synthetic wastewater. The proper trace element solution was added [16]. Influent COD_Cr_ was 800–1000 mg/L and NH_4_^+^-N was 55–60 mg/L.

Air diffusers supplied by air pumps provided a superficial gas velocity of 1.2–1.4 cm/s. The reactors were operated in successive cycles of 3 h including feeding of 5 min, aeration of 145 min, settling of 5 min before day 27 and 1 min after day 27, discharge and idle time of 7 min. The organic load was about 4 kg COD_Cr_/(m^3^·d). Thus, a feast-famine mechanism in sequencing batch process, short settling time and high shear force were applied to develop aerobic granular sludge.

The micropowder was made of the residual dry sludge from a sludge thickener of a local municipal sewage treatment plant located in the city of Hangzhou (China). The sewage sludge was dried in the oven for 12 h under 100 °C in a lab, then crushed with a multi-function grinder (XB-02, Xiaobao Electric Co. , Ltd., Yongkang city, China) to obtain 20–250 µm micropowder. Figure S1 shows the micropowder shape is irregular and rough.

Considering organic matters dissolved out from micropowder, variation of COD was tested in an SBR added 12 g micropowder and fed by fresh water. It was observed that COD value increased abruptly to 132 mg/L. The reason is the micropowder from the waste sludge contains organic matters that dissolved into fresh water.

The first experiment was comparison of aerobic sludge granulation in R1 (no addition of micropowder) and R2 (addition of 4 g micropowder), fresh activated sludge from a municipal wastewater treatment plant was inoculated when the reactors started. The goal of this experiment was to investigate whether the micropowder could shorten the aerobic granulation time.

The second experiment was comparison of aerobic sludge granulation in R1 (no addition of micropowder) and R2 (addition of 12 g of micropowder), bulking sludge from a reactor was inoculated when the reactors started. In the whole experiment, the time of addition of the micropowder in the first and second experiment was in both cases before the experimental started.

The third experiment was to observe the variation of sludge with overgrown filamentous bacteria in R1 after added 8 g of micropowder. The aims of the second experiment and third experiment were to investigate whether the bulking sludge (filamentous bacteria) could be controlled by adding micropowder. In this study, in each experiment micropowder was added once to reduce the impact on the MLSS by adding too much micropowder.

### 2.2. Analytical Methods

Chemical oxygen demand (COD_Cr_), ammonium (NH_4_^+^-N), mixed liquor suspended solids (MLSS) and the sludge volume index (SVI_30_) were assayed according to the standard methods [17]. The morphology of the sludge flocs and the aerobic granules were observed under an optical microscope (CX31, Olympus, Tokyo, Japan). The size of the aerobic granules was determined by an image-Pro Plus (Analysis 6.0, Olympus Soft Imaging Solution).

The elements of granular sludge and micropowder were analyzed by XRF spectrometry. Samples were dried at 110 °C for over 6 h in a drying oven and then dried in a muffle furnace at 600 °C for over 1 h before analysis. Micropowder (2 g) was dried in a muffle furnace at 600 °C for 2 h to analyze the proportion of inorganic and organic matters. The results showed that micropowder contained inorganic (62.5%) and organic matters (37.5%).

## 3. Results and Discussion

### 3.1. The Effect of Adding Micropowder on the Formation of Aerobic Granules

**Figure 1:**
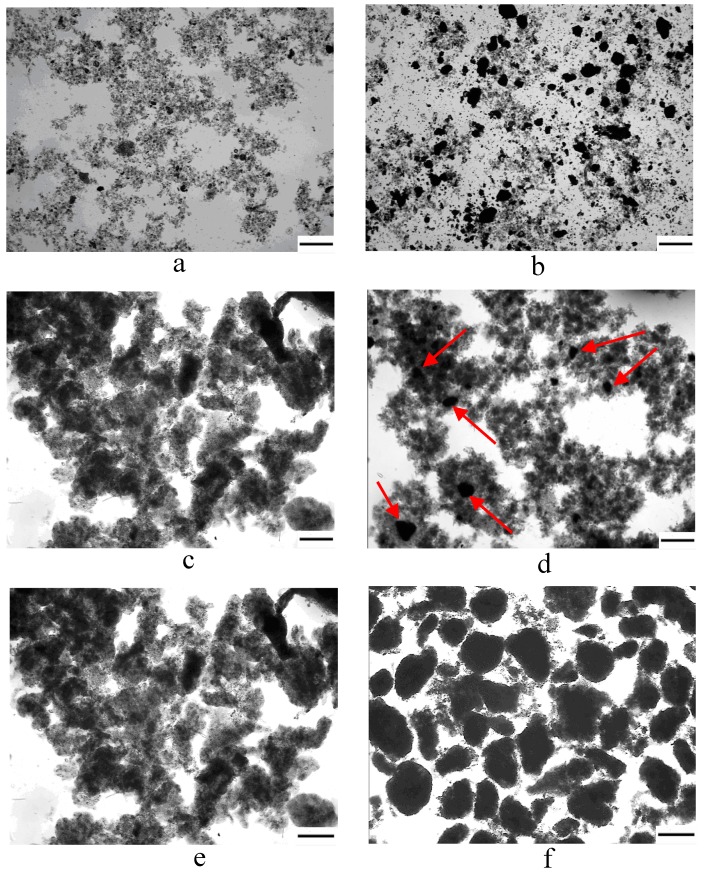
Sludge variation in R1 and R2 (**a**) inoculum; (**b**) inoculum and adding micropowder; (**c**) and (**d**) represent sludge on day 6 in R1 and R2; (**e**) and (**f**) represent sludge on day 30 in both reactors; the arrow (→) indicates the micropowder, scale bars: a, b c and d = 200 µm; e and f = 400 µm.

In the first experiment, inoculated sludge flocs and micropowder could be clearly distinguished in the reactors (Figure 1(a) and (b)). In R1, a few granules occurred on day 6 (Figure 1(c)) and loose and irregular granular sludge was observed on day 30 (Figure 1(e)). In R2, flocs were attached and aggregated gradually to the micropowder on day 6 (Figure 1(d)). Compared with R1, more compact and round granules were achieved in R2 on day 30 (Figure 1(f)). The results indicated that adding the micropowder had a positive effect on the formation of aerobic granules and shortening the time of aerobic granulation.

**Figure 2:**
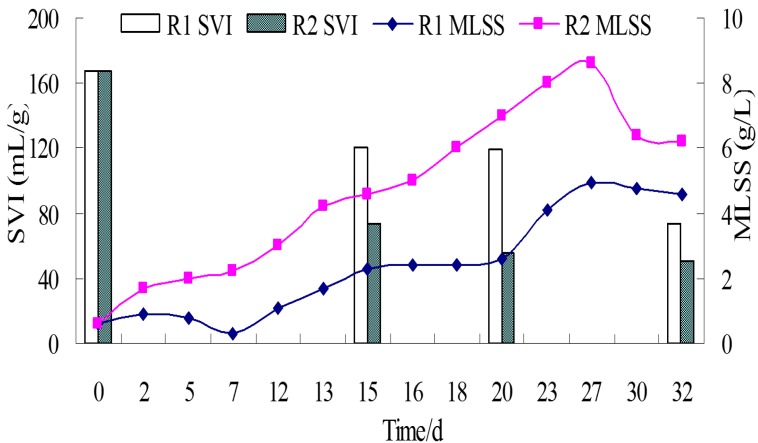
Variation of SVI and MLSS with time in R1 and R2.

The MLSS concentration of the two reactors increased gradually during the granulation process and reached maxima of 4920 mg/L and 8621 mg/L on day 27, respectively (Figure 2). Less sludge was washed out from R2 than R1 due to the faster sludge granulation in R2 when the settling time was shortened from 5 min to 1 min after 27 days, which resulted in MLSS in R2 being decreased at the end of the experiment. On day 15, the SVI of R1 and R2 were decreased from the initial 167 mL/g to 121 mL/g and 73 mL/g, respectively. On day 32, these decreased to 73 mL/g and 51 mL/g, respectively (Figure 2). The cross section of granules and crushed granules from the two reactors showed that micropowder existed in the granules (Figure S2), indicating that micropowder might act as nuclei and accelerate aerobic granulation.

**Figure 3:**
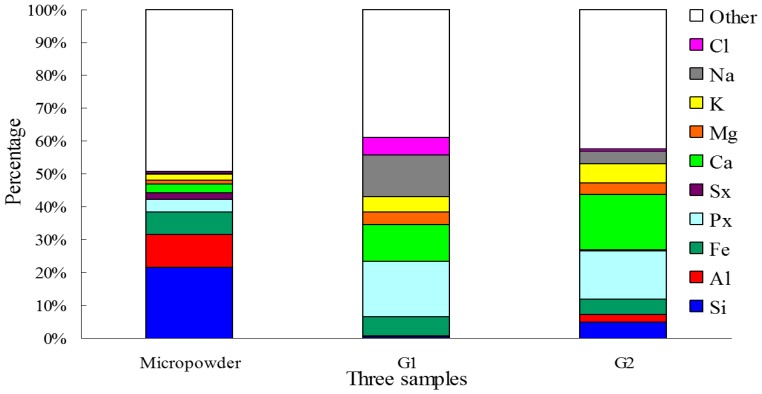
Elemental analysis of micropowder and granules by XRF, G1 and G2 respectively represent granules from R1 (no addition of micropowder) and R2 (adding micropowder) on day 24.

The elements of the micropowder and granules in both reactors were analyzed by XRF, demonstrating that Si (21.49%), Al (10.1%) and Fe (6.75%) were dominant in the micropowder (Figure 3). It was worth noting that the proportions of Si and Al of granules in R2 were 4.99% and 2.45% (Figure 3 G2 sample were 0.5% and 0.27% in R1 (Figure 3 G1 sample 

The result suggests that the Si and Al in the granules of R2 came from the micropowder because the raw wastewater contained no Si and Al. This implies that micropowder might act as nuclei in aerobic granulation. As shown in Figure S3, the reactor performance in terms of COD and NH_4_^+^-N removal indicates that R2 was better than R1, which might be related to the MLSS. The MLSS levels achieved were 13,534 mg/L and 2105 mg/L in R2 and R1, respectively.

### 3.2. Inducing and Forcing Filamentous Bacteria as Granule Framework by Adding Micropowder

**Figure 4:**
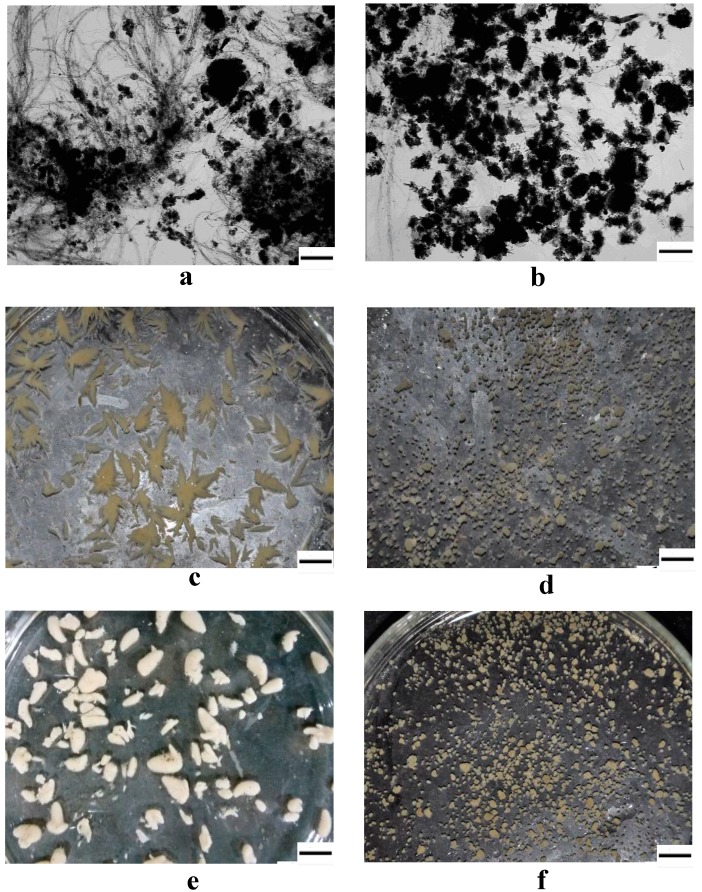
Images of sludge development in R1 (**a**, **c** and **e**) and R2 (**b**, **d** and **f**); (**a**), (**b**) and (**c**), (**d**) respectively represent sludge on day 7 and day 27; (**e**) indicates adding micropowder after 5 days in R1; (**f**) indicates granule in R2 on day 53; scale bar = 200 µm.

**Figure 5:**
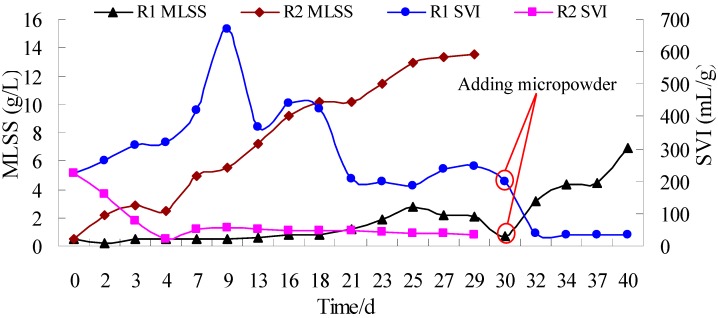
Variation of MLSS and SVI after inoculated high SVI sludge and adding micropowder.

In the secondary experiment, the high SVI (225 mL/g) sludge dominated by filamentous bacteria, which was aerated without any feeding for 3 days in a lab reactor, was used as the inoculated sludge for R1 and R2. The micropowder (12 g) was added to R2 before start up. More filamentous bacteria occurred in R1 after 7 days (Figure 4(a)). The filaments formed flower-like aggregates on day 27 (Figure 4(c)) and SVI was still high in R1 (Figure 5). In contrast, filamentous bacteria were controlled and formed granules in R2 by adding micropowder (Figure 4(b) and (d)).

In the third experiment, started on day 28 after the secondary experiment, sludge dominated by the filaments in R1 was aerated for 2 days without any feeding. On day 30, raw wastewater was fed again and 8 g of micropowder was added in R1 (Figure 5). After 5 days, the flower-like sludge had changed into large granules with a mean size of 4 mm (Figure 4(e)) and SVI had decreased to 37 mL/g (Figure 5). By comparison, the granules in R2 still remained stable after 53 days and the granules grew larger with a mean size of 550 µm (Figure 4(f)). The SEM photographs show the surface structure of inoculum and granules (Figure S4). An amount of filamentous bacteria was observed in the inoculum (Figure S4a). The filamentous bacteria became less on the granule surface in R2 because they were controlled by adding micropowder (Figure S4c). Denser and stronger filamentous bacteria were staggered on the surface of larger granules in R1 (Figure S4b). This implied that rapid formation of large granules needs an amount of filamentous bacteria as framework.

### 3.3. Characteristics of Micropowder for Sludge Granulation

The unique characteristics of this micropowder were proposed to be key for aerobic sludge granulation. First, this micropowder was taken from the residual sludge of a wastewater treatment plant and reused to accelerate activated sludge granulation, making it cheaper than other alternative materials.

Second, the size of this micropowder is controlled in a range from 20 to 250 µm in diameter, while cocci are around 0.5 to 1.0 μm in diameter, bacillus range from 2.0 to 5.0 μm in length, and filamentous bacteria range from 50 to 800 μm in activated sludge. It suggests that the micropowder is suitable as nuclei to induce bacteria attachment and aggregation.

Third, the micropowder was added in the reactor to rapidly raise the organic matter concentration due to its dissolving out. A higher organic load could control the growth of filamentous bacteria and be beneficial to aerobic granulation [18,19].

Fourth, the movement of this micropowder provides a special shear force to shape the microbial aggregates under the combined action of air and water flow, forcing the extended outside filamentous bacteria to shrink and frame granules [20].

**Figure 6:**
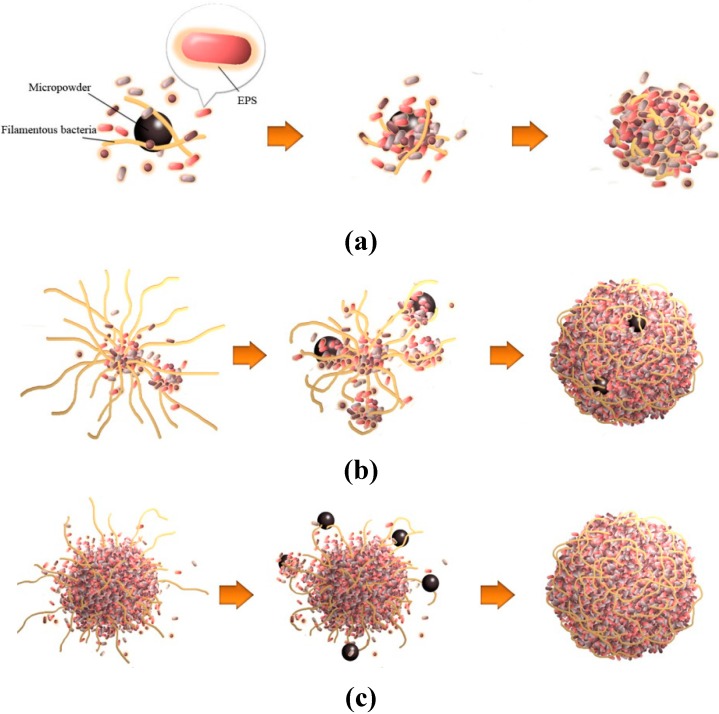
Mechanistic hypotheses to explain the acceleration of sludge granulation by adding micropowder: (**a**) Micropowder, EPS and filamentous bacteria respectively act as the nuclei, glue and framework for granulation; (**b**) Micropowder increases nuclei, organic load and shear force abruptly for starved sludge with overgrown filamentous bacteria acting as a framework to form large granules; (**c**) Micropowder friction restricts filamentous bacteria from extending outside and shapes granules.

### 3.4. Mechanistic Hypotheses for the Acceleration of Sludge Granulation by Adding Micropowder

The widely accepted mechanisms of aerobic sludge granulation are described by the nucleation hypothesis and selection pressure hypotheses [4,8,10]. In order to accelerate sludge granulation, adding dry sludge micropowder plays a positive effect. The mechanistic hypotheses proposed to explain this phenomenon can be summarized as follows:

(a) Adding the micropowder shortens sludge granulation time because the micropowder acts as nuclei to induce microbial aggregation. Simultaneously, the EPS of the activated sludge acts as glue [21] and filamentous bacteria act as the framework (Figure 6(a)) [22]. The micropowder size, ranging from 20 to 250 µm might match that of cocci, bacillus and filamentous bacteria for aggregation.
(b) Adding micropowder controls filamentous bacteria to shrink and frame granules because the micropowder not only acts as nuclei, but also increases the organic load and shear force abruptly for starved sludge. It is remarkable that adding micropowder in the reactor with overgrown filamentous bacteria rapidly leads to the formation of large and compact granules (Figure 6(b)).
(c) The micropowder, driven by airflow and liquid in the reactor, causes more collision and friction with microbial aggregates, thus playing an important role in shaping the surface of filamentous bacteria. This infers that adding micropowder restricts filamentous microorganisms from extending outside the granules (Figure 6(c)).

## 4. Conclusions

Adding dry micropowder made of waste sludge played an effectively role in accelerating aerobic granulation. The granule formation time was shortened by adding micropowder when the reactor started up. Even if the bulking sludge was inoculated with overgrown filamentous bacteria, large granules were also formed by adding micropowder. The micropowder is not only beneficial to accelerate aerobic granulation, but also uses recycled waste sludge for a lower overall waste treatment cost.

## Supplementary Files

Supplementary File 1

## References

[1] Liu Y., Tay J.H. (2004). State of the art of biogranulation technology for wastewater treatment. Biotechnol. Adv..

[2] Adav S.S., Lee D.J., Show K.Y., Tay J.H. (2008). Aerobic granular sludge: recent advances. Biotechnol. Adv..

[3] Khan M.Z., Mondal P.K., Sabir S. (2013). Aerobic granulation for wastewater bioremediation: a review. Can. J. Chem. Eng..

[4] Gao D.W., Liu L., Liang H., Wu W.M. (2011). Comparison of four enhancement strategies for aerobic granulation in sequencing batch reactors. J. Hazard Mater..

[5] Lettinga G., van Velsen A.F.M., Hobma S.W., de Zeeum W., Klapwijk A. (1980). Use of the upflow sludge blanket (USB) reactor concept for biological wastewater treatment especially for anaerobic treatment. Biotechnol. Adv..

[6] Yu H.Q., Fang H.H.P., Tay J.H. (2000). Effects of Fe^2+^ on sludge granulation in upflow anaerobic sludge blanket reactors. Water Sci. Technol..

[7] Ren T.T., Liu L., Sheng G.P., Liu X.W., Yu H.Q., Zhang M.C., Zhu J.R. (2008). Calcium spatial distribution in aerobic granules and its effects on granule structure, strength and bioactivity. Water Res..

[8] Liu L., Gao D.W., Zhang M., Fu Y. (2011). Comparison of Ca^2+^ and Mg^2+^ enhancing aerobic granulation in SBR. J. Hazard Mater..

[9] Zhou D.D., Liu M.Y., Gao L.L., Shao C.Y., Yu J. (2013). Calcium accumulation characterization in the aerobic granules cultivated in a continuous-flow airlift bioreactor. Biotechnol. Lett..

[10] Wan C.L., Lee D.J., Yang X., Wang Y.Y., Liu X. (2015). Calcium precipitate induced aerobic granulation. Bioresour. Technol..

[11] Zhang Y., An X., Quan X. (2011). Enhancement of sludge granulation in a zero valence iron packed anaerobic reactor with a hydraulic circulation. Process Biochem..

[12] Kong Q., Ngo H.H., Shu L., Fu R.S., Jiang C.H., Miao M.S. (2014). Enhancement of aerobic granulation by zero-valent iron in sequencing batch airlift reactor. J. Hazard Mater..

[13] Pijuan M., Werner U., Yuan Z.G. (2011). Reducing the startup time of aerobic granular sludge reactors through seeding floccular sludge with crushed aerobic granules. Water Res..

[14] Verawaty M., Pijuan M., Yuan Z.G., Bond P.L. (2012). Determining the mechanisms for aerobic granulation from mixed seed of floccular and crushed granules in activated sludge wastewater treatment. Water Res..

[15] Li A.J., Li X.Y., Yu H.Q. (2011). Granular activated carbon for aerobic sludge granulation in a bioreactor with a low-strength wastewater influent. Sep. Purif. Technol..

[16] Li J., Garny K., Neu T., He M., Lindenblatt C., Horn H. (2007). Comparison of some characteristics of aerobic granules and sludge flocs from sequencing. Water Sci. Technol..

[17] APHA (2006). Standard Methods for the Examination of Water and Wastewater.

[18] Show K.Y., Lee D.J., Tay J.H. (2012). Aerobic granulation: Advances and Challenges. Appl. Biochem. Biotech..

[19] Yang Y.C., Liu X., Wan C.L., Sun S.P., Lee D.J. (2014). Accelerated aerobic granulation using alternating feed loadings: Alginate-like exopolysaccharides. Bioresour. Technol..

[20] Liu Y., Liu Q.S. (2006). Causes and control of filamentous growth in aerobic granular sludge sequencing batch reactors. Biotechnol. Adv..

[21] Liu Y.Q., Liu Y., Tay J.H. (2004). The effects of extracellular polymeric substances on the formation and stability of biogranules. Appl. Biochem. Biotech..

[22] Morgenroth E., Sherden T., Van Loosdrecht M.C.M., Heijnen J.J., Wilderer P.A. (1997). Aerobic granular sludge in a sequencing batch reactor. Water Res..

