# Supplementary material for: Accelerating Aerobic Sludge Granulation by Adding Dry Sewage Sludge Micropowder in Sequencing Batch Reactors
Source: Int J Environ Res Public Health. 2015 Aug 21;12(8):10056–65. doi: 10.3390/ijerph120810056 (PMC4555328; doi:10.3390/ijerph120810056)
Supplement: Supplementary File 1 [file ijerph-12-10056-s001.pdf]

Supplementary Information

Accelerating Aerobic Sludge Granulation by Adding Dry Sewage Sludge Micropowder in Sequencing Batch Reactors

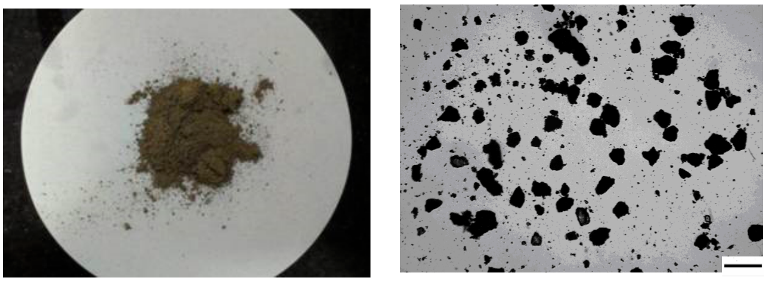

(**a**) (**b**)

Figure S1. Images of micropowder by camera (**a**) and microscope (**b**), scale bar = 200 μm

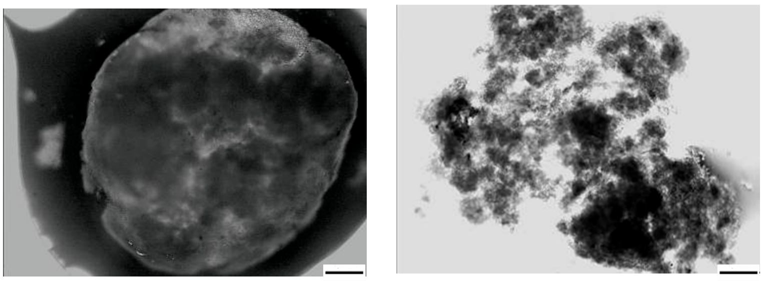

(**a1**) (**a2**)

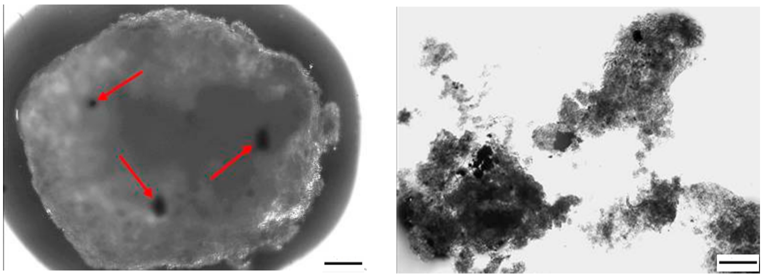

(**b1**) (**b2**)

Figure S2. Comparison of granules from R1 and R2: (**a1**) and (**b1**) represent cross section of granules from R1 and R2; (**a2**) and (**b2**) represent rushed granules from R1 and R2; the arrow (→) indicates the micropowder, scale bar = 200 μm.

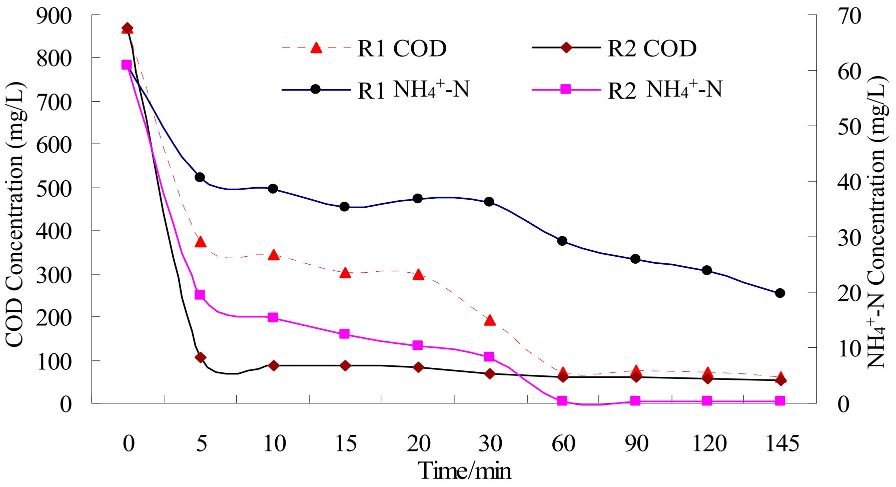

Figure S3. Variation of COD and NH_4^+-N concentration in R1 and R2 of a circle.

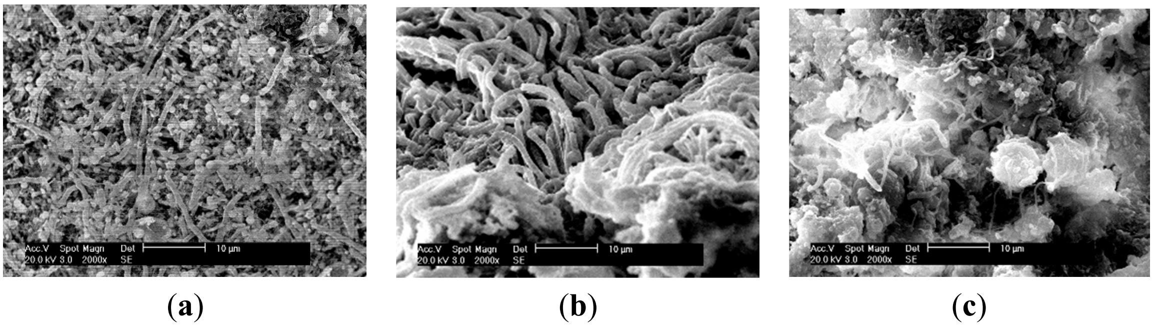

Figure S4. SEM photographs of the inoculum (**a**), aerobic granules from R1 (**b**) and R2 (**c**) at day 35.

© 2015 by the authors; licensee MDPI, Basel, Switzerland. This article is an open access article distributed under the terms and conditions of the Creative Commons Attribution license (http://creativecommons.org/licenses/by/4.0/).
